# HTLV-1 infection of myeloid cells: from transmission to immune alterations

**DOI:** 10.1186/s12977-019-0506-x

**Published:** 2019-12-23

**Authors:** Brenda Rocamonde, Auriane Carcone, Renaud Mahieux, Hélène Dutartre

**Affiliations:** 1 International Center for Research in Infectiology, Retroviral Oncogenesis Laboratory, INSERM U1111 - Université Claude Bernard Lyon 1, CNRS, UMR5308, Ecole Normale Supérieure de Lyon, Université Lyon, Lyon, France; 2 Equipe labelisée par la Fondation pour la Recherche Médicale, Labex Ecofect, Lyon, France

**Keywords:** HTLV-1, Myeloid cells, Infection

## Abstract

Human T cell leukemia virus type 1 (HTLV-1), the etiological agent of adult T-cell leukemia/lymphoma (ATLL) and the demyelinating neuroinflammatory disease known as HTLV-1-Associated Myelopathy/Tropical Spastic Paraparesis (HAM/TSP), was the first human retrovirus to be discovered. T-cells, which represent the main reservoir for HTLV-1, have been the main focus of studies aimed at understanding viral transmission and disease progression. However, other cell types such as myeloid cells are also target of HTLV-1 infection and display functional alterations as a consequence. In this work, we review the current investigations that shed light on infection, transmission and functional alterations subsequent to HTLV-1 infection of the different myeloid cells types, and we highlight the lack of knowledge in this regard.

## Background

Human T-cell leukemia virus type 1 (HTLV-1) was the first retrovirus known to be associated to a neoplastic disease, a type of T-cell leukemia in humans. In early 80’s, several works described HTLV-1 endemicity in Japan, Caribbean, South America, and central Africa [1]. Later, it was also shown that the virus was endemic in Australian aborigines [2]. Even though most infected individuals will remain asymptomatic carriers (ACs), between 2 and 5% of the carriers will develop adult T-cell leukemia/lymphoma (ATLL) [3]. HTLV-1 infection remains latent 20–30 years before ATLL development. Life expectancy of patients suffering from the most aggressive ATLL form remains below 12 months [4]. The acute phase is characterized by the presence of an elevated number of HTLV-1-infected CD4^+^CD25^+^ T-cells in blood [5, 6]. HTLV-1 infection is also associated with HTLV-1-Associated Myelopathy/Tropical Spastic Paraparesis (HAM/TSP), a neuroinflammatory disease that arises in 1–3% of all HTLV-1 infected individuals. HAMP/TSP leads to demyelination middle-to-lower thoracic cord, resulting in motor dysfunction of the lower limbs [7, 8]. The hallmark of the HAM/TSP pathology is represented by parenchymal lymphocytic cell infiltration at the level of the lower thoracic spinal cord [9, 10]. Besides an increased proviral load (PVL), which is observed in patients with ATLL or HAM/TSP [11], these two diseases seem not only driven by the oncogenesis properties of HTLV-1 itself but also by potential interactions between the virus and its host’s immune system, although the complete mechanisms leading to HAM/TSP or ATLL development are not fully understood.

A number of inflammatory diseases such as uveitis [12, 13], arthropathy, pneumopathy, dermatitis, exocrinopathy and myositis [14, 15] have also been shown to be linked to HTLV-1 infection.

CD4^+^, and to a lesser extent CD8^+^ T-cells represent the main target of HTLV-1 in vivo, and they present the highest PVL [11, 16]. Therefore, deciphering the role of T-cells in disease progression has been the focus of many research teams during the past decades. This has allowed researchers to understand uninfected T-cells ability to respond to infection, to be activated, or infected and/or transformed by HTLV-1. Both ATLL and HAM/TSP diseases were shown to be linked to gene expression deregulation, increased expression of pro-inflammatory cytokines such as Tumor necrosis factor alpha (TNF-α) and Interferon gamma (IFN-γ) and increased Interleukine 12 (IL-12) levels. This maintains T-cells in a proliferative state [17, 18] and potentially influences the pathology and clinical manifestations of the end-stage disease. Although instrumental for understanding, classifying and characterizing HTLV-associated diseases, this “T-cells focused” research did not allow scientists to fully understand how HTLV-1 spreads within newly infected individuals, and why infection can lead to two immunological opposite diseases. Furthermore, currently used therapeutic strategies targeting adaptive immune response have shown limited efficiency [19, 20]. Finally, T-cell focused-studies did not explain why a small fraction of infected people would develop diseases, while the vast majority will remain asymptomatic. Altogether, these data highlight the fact that, besides CD4^+^ T-cells, HTLV-1 infection may affect other cell types, and that T-cells functional alteration may be the top of the iceberg, as a result of earlier or subtler modifications of others cells types or immune compartments. Since it is at the forefront of induction and maintenance of immune responses, myeloid compartment may deserve a special interest, through its unique ability to polarize naïve T-cells into either cytotoxic, inflammatory, regulator or tolerant T-cell effectors [21–25]. Thus, myeloid cells may interact with HTLV-1 throughout the course of infection, during the acute and/or chronic phases, either as potential targets of HTLV-1 infection or because there are functionally altered.

In this work, we review the current literature investigating the role of myeloid cells during HTLV-1 infection, and we highlight the lack of knowledge that impairs researchers from fully understanding HTLV-1 infection and, potentially, the differential mechanism of disease evolution.

## Infection of myeloid cells by HTLV-1

### In vivo

**Fig. 1:**
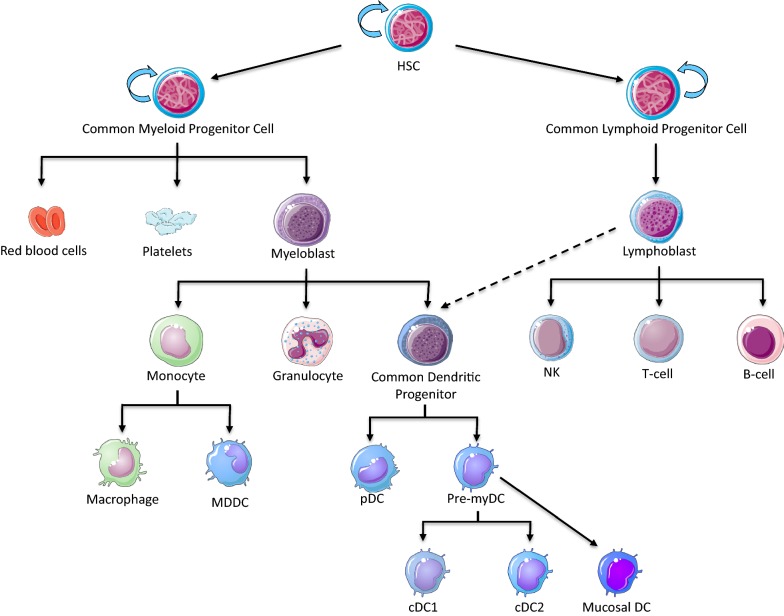
Hematopoietic stem cells are multipotent progenitors capable of give rise to both myeloid and lymphoid cell lineages. Myeloid cells derive from a common myeloid progenitor lineage whose differentiation give rise to several cell-types such as myeloid and plasmacytoïd dendritic cells found in blood, mucosal dendritic cells found in skin, lung, or intestine mucosa, and monocytes that can alternatively further differentiate into macrophages or dendritic cells in tissues upon injury

Myeloid cells derive from a common myeloid progenitor whose differentiation gives rise to several cell-type forming the myeloid compartment. This includes dendritic cells present in blood (named as myeloid or myDC throughout the text) and in the different mucosa, plasmacytoid dendritic cells (pDC), and monocytes, that can further differentiate into macrophages or dendritic cells (Fig. 1). As sentinel cells, DCs are present in all mucosa (i.e. intestine, vaginal or lung) and in circulating blood [26, 27]. Due to its mode of transmission i.e. breast feeding, sexual intercourse or blood transfusion, HTLV-1 might interact with DCs during primo infection. Indeed, infection of blood dendritic cells was first reported in 1992 through detection of viral DNA in DCs purified from HTLV-1 infected individuals blood, using in situ hybridization [28]. Viral DNA was thereafter also detected by PCR in monocytes from HAM/TSP patients [29, 30], in pDC from HTLV-1 infected asymptomatic individuals [31], and in macrophages from milk obtained from infected mothers [32]. Of note viral DNA was not searched in any mucosal DC, although these cells might be in contact with HTLV-1 during transmission through breast feeding or unprotected sexual practices (see Table 1).

**Table 1:** Recapitulation of the susceptibility of in vitro and in vivo infection, the capacity for viral transmission and the immune response adopted by the different cell types upon HTLV-1 infection

Cell type/condition	Mucosal DC (TGF-β derived)	MDDC (IL-4 derived)	myDC	pDC	Monocyte	Macrophage	HSC
In vitro infection	+	+	+	±	–	?	?
In vivo infection	?	n/a	+	+*	+*	+	+
In vitro transmission	?	+	+	±	–	?	?
Ex vivo immune response	?	CD80, CD86, CD83, MHCII IL-12, TNFα, Mip1β	?	IFNα	TNFα, IFN-I CD16	CCL5, CXCL9, CXCL10, TNFα, IFN-I IL-10	?

n/a: non applicable. Asterisk (*) indicates the presence of viral DNA originating from HSC differentiation

Recently, HTLV-1 DNA was also detected in hematopoietic stem cells (HSC) in vivo [33, 34]. HSC are multipotent, self-replicative blood cells able to give rise to both myeloid and lymphoid lineages during hematopoiesis occurring in the bone marrow (Fig. 1). Using cutting-edge molecular biology tools, HSC-derived blood cells (neutrophils, monocytes, B cells, CD8^+^ and CD4^+^ T-cells) isolated from HAM/TSP blood samples were shown to share the same HTLV-1-integration site. Same results were obtained using cells from STLV-1 infected Japanese macaques. These results demonstrate a primary infection of stem-cell lineage [33, 34].

### In vitro

To validate these in vivo results, several groups performed in vitro exposure of blood macrophages, myDC, monocytes, monocytes derived DC (MDDC) or pDC to HTLV-1 [29, 35–43]. Dendritic cells-derived from monocytes generate larger amount of DC, compared to DC purified from blood or extracted from mucosa, that are furthermore transcriptionally and functionally close to myeloid DC [44]. Using different cocktail of cytokines, monocytes can generate different DC subtypes, defined as surrogates of mucosal DC (when differentiated in the presence of Transforming growth factor beta, TGF-β), myeloid blood DC (when differentiated in the presence of IL-4) or activated/mature DC (when differentiated in the presence of IFN-I). Using these experimental settings, it was shown that both TGF-β DC [39, 43, 45] and IL-4 DC [29, 38, 42, 43] were susceptible to HTLV-1 infection (see Table 1), while IFN-DCs were resistant [43]. Similar expression of HTLV-1 receptor, i.e. Glut-1 and BDCA-4/NRP-1 was observed in susceptible and resistant DCs, and consequently viral entry, measured by flow cytometry using p19gag intracellular staining, was not lower, but in contrast increased in resistant DC [43]. Resistance of IFN-treated DC to HTLV-1 infection was not due to the presence of exogenous recombinant IFN-α, since, in contrast to T-cells results [46, 47] treatment of IL-4 DC with recombinant IFN-α did not prevent their infection [43]. On the contrary, DC maturation account for their resistance to HTLV-1 infection [43]. These results suggest that restriction factors different from those induced by IFN treatment might be induced during DC maturation and might be responsible for their resistance to HTLV-1 infection. Finally, comparison of MDDCs generated using different cytokine cocktails revealed that MDDCs generated in presence of IL-4 were more susceptible to HTLV-1 infection than those generated in the presence of TGF-β, with again similar HTLV-1 receptor expression and equivalent HTLV-1 entry in DC generated in presence of IL-4 or TGF-β [43]. In addition, IL-4 DCs are more susceptible to HTLV-1 infection than their autologous T-cells counterparts [42]. DC infection was confirmed using myeloid DC purified from blood [39]. Altogether these data support the idea that DC but not T-cells, might be the first cell encountered by HTLV-1 during the primo infection [48], independently of the route of infection, i.e. bloodborne or mucosal. Nevertheless, why different subtypes of DCs are differentially susceptible to HTLV-1 infection is still not fully understood.

In contrast to MDDC, investigations using pDC represented a challenge due to their sparse representation in human blood. Nevertheless, one study reported in vitro infection of blood pDC by cell-free HTLV-1 viral particles, with a permanent viral production even after several week of pDC culture [39]. This result was very surprising given the fact that pDCs have a half-life of 72 h in culture and in vivo [49, 50] and more importantly, because they are commonly known as fully resistant to any viral infection due to their strong ability to produce type I interferon (IFN-I) upon pathogens sensing [51]. These results were not reproduced recently, when pDC were exposed to HTLV-1-infected cells lines instead of cell free virus (see below) and were cultured for a short period of time consistent with their in vivo lifetime. In this report, no viral infection was observed, as determined by absence of viral Tax expression, a viral protein that is absent from the incoming viral particle [52].

While several groups were able to show in vitro infection of MDDC [36, 38–43, 53, 54] and of macrophages [35, 55], in vitro infection of monocytes was less documented [29, 37, 54] and seems more controversial. Indeed, while in vitro infection was observed using either adherent plastic monocytes infected with cell-free HTLV-1 [29], or the monocytic-like cell line THP-1 after co-culture with B-cell lines previously transfected with HTLV-1 molecular clone [37], it was not observed when using purified monocytes exposed to highly concentrated cell-free HTLV-1 [54]. Interestingly, all these studies detected expression of the viral protein Gag, either by flow cytometry 5 days post infection [29], by ELISA using culture supernatant 10 days post-infection [37] or western-blot 48 h post-infection [54], suggesting that (i) viruses might persist for long period of time in monocytes or monocytes-like cell lines and that (ii) viral Gag detection should not be used as a tool to conclude for productive HTLV-1 infection. Indeed, Tax expression was not observed in purified monocytes [54], but was not investigated in plastic adherent monocytes infection [29]. In addition, it was further reported that HTLV-1 exposure of monocytes led to their apoptosis, because of reverse transcription inhibition by SAM domain and HD domain-containing protein 1 (SAMHD-1). This led to Stimulator of interferon genes (STING)-signaling dependent sensing of viral replication intermediates [54]. Thus, it seems likely that primary monocytes might not be productively infected per se in vitro by HTLV-1. Productive infection of monocyte-like cell line could result from defective SAMHD-1 or STING signaling, due to leukemic transformation, and might not reflect the in vivo situation, although this remains to be determined.

**Fig. 2:**
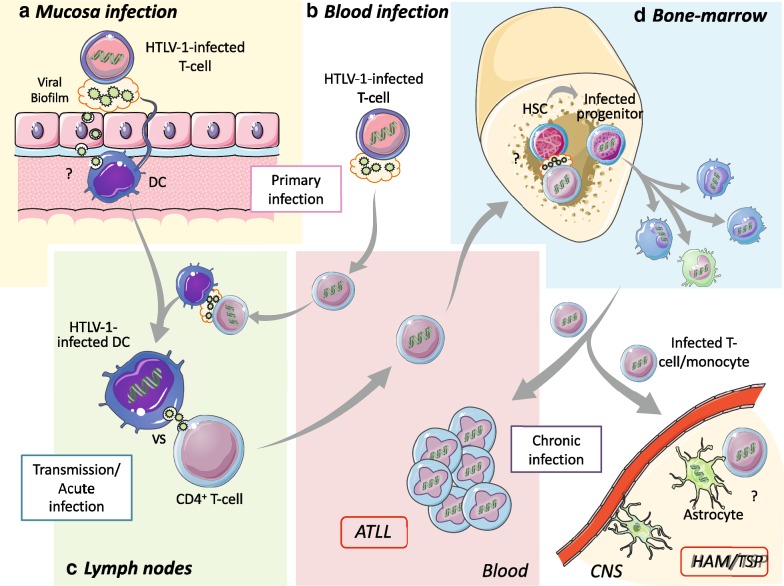
Schematic model of HTLV-1 transmission to new individuals during primary infection and after exposure to donor infected T-cells or macrophages (Takeuchi [32], de Revel [55]). Infected T-cells from infected donors are colored in dark blue. Viral expression is depicted by viral particles at the surface of infected T-cells in biofilm (represented as a cloud) or in infected DC. Viral infection is depicted as DNA present in nucleus. **a** After contact of donors infected cells with recipient DC present in the genital track and intestine mucosa, recipient mucosal dendritic cells could be at the forefront of the infection and being the first to be de novo infected probably by viral biofilm. **b** After transfer of donor infected cells through blood, donor infected T-cells might transit to lymph node, in which they could transfer HTLV-1 to naïve resident DC. **c** In lymph nodes, productively infected DC could contact naïve T-cells and concomitantly transfer HTLV-1 to T-cells through viral synapse. **d** Recipient infected DC might then migrate to bone marrow in which they could infect HSC. Bone marrow hematopoiesis will results in HSC differentiation and viral dissemination in multiple cell type that may have not directly contacted newly produced HTLV-1 particles. Inherited viral DNA would increase PVL and may disseminate HTLV-1 to CNS

Since in vitro infection is abortive, how viral DNA could be detected in monocytes and pDC after their purification from patient’s blood remains to be understood. One possible explanation relies on the fact that viral DNA was detected in HSC [33, 34]. The fact that an identical viral integration site was identified in monocytes, pDC and HSC from a given individual, demonstrated that the viral DNA present in monocytes or pDC has been inherited from infected HSC. The mechanism of HSC infection by HTLV-1 has not been investigated yet. It looks reasonable to hypothesize that infected T-lymphocytes trafficking in the bone marrow during primary infection may lead to infection of bone-marrow resident HSCs [56]. Later on, infected HSCs, will give rise to myeloid and lymphoid lineage cell types, thus spreading the infection (Fig. 2).

### Viral transmission to T-cells by myeloid cells

As expected, all in vitro infected DCs were shown to produce HTLV-1 viral particles, detected in the supernatant using Gag p24 detection kit [39] or in cell cytoplasm using imaging after p24 immunostaining [41] or using flow cytometry after Tax immunostaining [43]. Productively infected DC can transmit HTLV-1 to T-cells [39, 42, 43]. Viral transmission was strongly impaired when DC were treated with antivirals such as Zidovudine (AZT) [39, 42] or when DC were matured using Toll-like receptor (TLR) agonist before their exposure to HTLV-1 [43]. Since HTLV-1 entry into these cells was not impaired regardless of their treatment [43], these results suggest that, in contrast to the human immunodeficiency virus (HIV) [57] HTLV-1 transmission from DC to T-cells requires first a productive replication. However, it is worth noting that, in some case, i.e. when HTLV-1 accumulated at MDDCs surface without internalization and thus without productive infection, viral transfer and productive T-cells infection was efficient [58].

The role of DC infection in HTLV-1 dissemination is also supported by investigations performed in animals. After dendritic cells depletion, mice infection with a chimeric HTLV-1 virus pseudotyped with the murine leukemia virus (MLV) envelop showed lower HTLV-1 proviral load in CD4^+^ T-cells, compared to a similar experiment performed in mice without DC depletion [59]. In addition, HTLV-1 viruses harboring mutations in the p12 and p30 regulatory genes were shown to be unable to infect dendritic cells in vitro and led to poor seroconversion rates of infected macaques [36]. Thus, in addition to be the first cells encountered by HTLV-1, DC might also be important intermediaries for viral dissemination and further T-cell infection. HTLV-1 will thereafter replicate through clonal expansion of infected T-cells.

### Mechanisms/route of viral transmission

HTLV-1 transmission requires an interaction between the target cell and an infected cell. Transfusion of cellular blood components of HTLV-1 infected donors results in infection of recipients, whereas recipients of non-cellular blood compartment are not infected [60, 61]. Importantly, 1- or 2-LTR DNA circles, that are  of an active HTLV-1 replication, are detected in the blood obtained either from asymptomatic HTLV-1 carriers, and from ATLL or HAM/TSP patients [62]. Thus this strongly suggests that true viral replication is maintained during infection. Indeed, a minimal number of 90,000 infected cells was estimated to be required for the infection of a given recipient [63]. Thus, infected cells are the infectious entity accounting for HTLV-1 transmission, and viral reactivation in donor-infected cells must occur during their transmission to new recipients. Given the viral latency of infected T-cells present in the blood of infected patients [64], and unless ex vivo peripheral blood mononuclear cells (PBMCs) from infected patients are cultured in vitro [65–68], it remains to be determined how DCs might be infected in vivo.

In vivo observations were confirmed by in vitro studies showing that cell–cell contact was required for HTLV-1 transmission between T-cells, either through (i) viral synapse formation between infected T-cells and target T-cell [69], (ii) the transfer of viruses accumulated at the surface of infected-cell and embedded in the extracellular matrix (ECM) of the viral biofilm, [42, 52, 70], or (iii) the transfer of virus through nanotubes induced in the infected cells by the p8 and p12 accessory viral proteins [71, 72] or Tax [73, 74]. Strikingly, DCs were first shown to be infected by highly concentrated cell-free HTLV-1 harvested in the supernatant of chronically-infected cell lines cultured at high density [39, 40]. These results challenged the idea that cell-free HTLV-1 was not infectious compared to cell–cell contact, as demonstrated in T-cells [42]. One possible explanation could be that viral biofilm was co-purified by accident and therefore cell-free preparation was not truly composed of cell-free virus but also of contained viral biofilm detached from infected cells and released in culture supernatant. This explanation is supported by the fact that HTLV-1 chronically infected cell lines lacking biofilm are poorly able to transfer HTLV-1 to other cells [70]. Consistent with this finding, HTLV-1 biofilm artificially separated from the surface of infected-cells was shown to efficiently infect MDDC and autologous T-cells. On the opposite, cell-free HTLV-1 viral preparation used in similar amount was not [42]. Altogether, this data thus demonstrated that as T-cells, MDDC, are not efficiently infected by cell-free HTLV-1. Rather, HTLV-1 biofilm present either at the surface of infected cells during cell–cell contact or in culture supernatant when cell culture is prolonged at high cell density is the efficient mode of transmission.

In the context of breast-feeding or sexual intercourse, infected T-cells present in the maternal milk or in the semen are exposed to the luminal side of intestine or genital mucosa, mainly composed of epithelial cells. However, HTLV-1 infected T-cells were shown to be unable to infect in vitro epithelial cells or to cross the epithelial barrier [45], thus precluding the hypothesis that they would contact DC for in vivo HTLV-1 transmission. In contrast, epithelial barrier was also shown to be pervious to HTLV-1 particles thanks to their transcytosis properties. Indeed, epithelial cells can capture HTLV-1 from infected T-cells present at the apical face and release the virus at the basal face using transcytosis [45]. It was further shown that after transcytosis through epithelial cells, HTLV-1 could infect MDDC placed underneath the epithelial barrier [45]. Interestingly in that case, cell–cell contact was still maintained since MDDC were adherent to epithelial cells. Whether epithelium could transfer viral biofilm using transcytosis was not addressed in that study. Alternatively, because DC generate long cellular protrusions to patrol the luminal environment, they might be infected at the edge of the protrusion by direct cell–cell contact with infected T-cells present in the lumen, without the need for infected T-cells or HTLV-1 biofilm to cross the epithelial barrier.

Altogether a model in which dendritic cells play a central role during primo-infection for HTLV-1 dissemination in new individuals can be proposed. In the case of breast-feeding or sexual transmission (Fig. 2a), capture and transcytosis of HTLV-1 particles by epithelial cells to underneath DC or DC infection after direct contact with luminal infected T-cells at the edge of DC protrusions, results in mucosal DC infection thus being the first de novo infected cell. Migration of infected DC to lymph nodes will then allow HTLV-1 transfer to T-cells (Fig. 2c). In case of contact with blood containing infected cells (Fig. 2b), low density of myeloid DC and high blood flow in recipient would very likely decrease the probability of donor-infected cell to contact recipient circulating DC. However, donors infected T-cells might transit to lymph nodes in which fluid circulation might be lower, density of dendritic cells higher, and viral latency relieved because of changes in nutrient availability [75]. This will allow viral expression in donor T-cells, their contact with recipient DC, subsequent viral transmission to DC, viral replication in DC and then final transmission to T-cells from productively infected DC (Fig. 2c).

Although not truly infected by HTLV-1, monocytes bearing viral DNA inherited from HSC differentiation [33, 34] may also participate in viral dissemination, especially to the central nervous system (CNS). Interestingly, HTLV-1 is latent in monocytes, as in T-cells, but can be reactivated after in vitro culture [76], although a mechanism that controls this viral reactivation has not been investigated yet. Indeed, monocytes can be found in the meninges and the choroid plexus [77–81] and play an essential role in CNS recovery upon injury or demyelinating diseases [82–84]. Although the mechanism is not well understood and as in T-cells, latent HTLV-1 in monocytes can be reactivated under certain conditions in vitro [76, 85]. Infiltration of silently infected monocytes in the CNS could be responsible, after viral reactivation, in viral dissemination to neural cells, in particular astrocytes [86]. Thus this will participate in the inflammatory loop characteristic of HAM/TSP [87] (Fig. 2d).

## Impaired function of HTLV-1 infected myeloid cells

Besides its viral transmission to T-cells, HTLV-1 presence and expression in myeloid cells may alter their function. However, compared to what is known regarding T-cells functional perturbation little is known concerning these changes [88]. Moreover, most studies have been focused on monocytes, investigating their frequencies or abilities to differentiate into DC. Very little is known concerning macrophages or pDC functions in HTLV-1 infected patients (see Table 1).

### Macrophages present altered cytokines production in HTLV-1-infected patients

Little is known about modifications in macrophage’s cytokines secretion upon HTLV-1 infection. Balistrieri et al. [89] demonstrated that monocytes-derived macrophages secreted important amounts of CC-chemokines when exposed to Tax. Moreover, upon stimulation, but also at steady state, macrophages from HTLV-1 infected patients (both asymptomatic carriers and HAM/TSP subjects) secrete elevated quantities of chemokines (C–C motif) ligand 5 (CCL5) and chemokine C-X-C motif ligand 9 (CXCL9), both acting as T-cell chemoattractant induced by IFNγ, and reduced quantities of anti-inflammatory cytokine IL-10, (Table 1) [90]. Furthermore, the levels of CXCL10 and TNF-α secretion were correlated with HTLV-1 proviral load. However, macrophages from HTLV-1-asymptomatic carriers and HAM/TSP were not impaired in their ability to kill intracellular pathogens.

### Patients monocytes frequencies, phenotype and functions are perturbed

Nascimento et al. [91] observed that monocytes from HTLV-1-infected individuals showed a decreased percentage of intermediate monocytes (CD16^+^CD14^+^) compared to monocytes from healthy donors, while the frequency of classical monocytes (CD16^−^CD14^+^) was not affected. Of note, frequency of non-classical monocytes (CD16^+^CD14^−^) was not investigated in this study. However, others using more precise identification of monocytes showed that frequency of non-classical (CD16^+^CD14^−^) monocytes was increased, while the frequency of classical monocytes was decreased in HTLV-1 infected individuals (AC and HAM/TSP) [30]. Interestingly, CD16^+^ monocytes (comprising both intermediate and non-classical) have been reported to be more prone to differentiation into dendritic cells and could be the monocyte subset committed to DC differentiation in vivo.

Direct alteration of monocytes from HAM/TSP patients was also reported and was associated with an increased ability to induce CD8^+^ T-cells degranulation compared to monocytes from healthy donors or from asymptomatic carriers [76, 85]. This property was linked to higher frequencies of monocytes expressing HLA-DR and CX3CR1 and producing TNF-α and IL1β after short in vitro culture [85] (Table 1). Interestingly, upon co-culture, these activated monocytes stimulate viral expression in CD4^+^ infected T-cells, suggesting that direct interaction of activated monocytes (including those that carry viral DNA) with CD4^+^ lymphocytes potentiate reactivation of viral replication, thus skewing monocyte immune function in favor of viral replication.

Yet the mechanism responsible of such monocytes activation is not known. Furthermore, whether these defects are linked to their proviral load in vivo is unknown, although a negative correlation between virus burden in intermediate monocytes and their phagocytic function was observed [30]. Alternatively, Matsuura et al. [92] observed that infected cells from HAM/TSP patients were able to transfer Tax to monocytes after close cell–cell contact in culture. This was then correlated to elevated numbers of CTL:CD14^+^ conjugates in samples from HAM/TSP patients compared to uninfected samples, and to the loss of monocytes populations after 18 h culture of PBMCs from HAM/TSP patients [92]. This suggests that monocytes from HAM/TSP patients can acquired Tax and then be targets for cytotoxic depletion by CD8+ T-cells, thus reducing monocytes count independently of their ability to be infected by HTLV-1 or to be differentiated in DC.

### Ability of monocytes from patients to differentiate into DC

Study performed in monocytes obtained from ATLL patients showed they poor ability to differentiate into Monocytes Derived Dendritic Cells in vitro, probably as a result in alterations of the CD16^+^ monocyte compartment by HTLV-1, as discussed above. Furthermore, MDDC derived from monocytes of ATLL patients have a reduced ability to present antigen and have altered capacities to stimulate proliferation of allogenic T-lymphocytes [29, 93]. In contrast, MDDCs obtained from HAM/TSP patients have increased capabilities to stimulate proliferation of autologous CD4^+^ and CD8^+^ T-lymphocytes [38], although their differentiation into MDDCs is also altered, with a lower expression of CD83, CD86 and CD1a [38, 91] (Table 1). Finally, differentiation defects of MDDCs from HAM/TSP patients is not due to their infection [85, 91], leaving opened the mechanism of these alterations.

Alternatively, impaired DC differentiation and decreased T-cell activation ability could be the consequence of an altered micro-environment in which monocytes originated. Interestingly, elevated levels of IL-10 were found in ATLL patients sera [94], whose production may be due to both HTLV-1 infected cells and surrounding micro-environment. DC development in the presence of IL-10 and TGF-β, can lead to tolerance and immune evasion. In addition, ex vivo, spontaneous TNF-α and IL-1β production by HAM/TSP patients monocytes impairs DC differentiation [85]. Finally, monocytes dysfunction or decreased ability to differentiate into MDDC could also be due to viral proteins (p8/12 p30 and Tax, see below) either directly expressed by monocytes containing viral DNA or delivered to monocytes after contact with infected cells. These hypothetical models now need to be confirmed in vivo.

### In vitro MDDC alterations induced by ectopic expression of p8/12, p30 or Tax

The viral proteins, p8, p12, p30 and Tax have been shown to be delivered from HTLV-1-infected cells to target cells by respectively nanotubes [72, 95, 96] and exosomes [97]. Furthermore, in in vitro studies using ectopic expression in cells from healthy donors, Tax has been shown to modulate class-I major histocompatibility complex (MHC-I) expression [98] and nuclear factor-kappa B (NFκB) signaling in T-cells [99], while p8/12 and p30 have been shown to modulate TLR4 expression [100] and TLR3/4 signaling in monocytes and MDDC [37]. Interestingly, although the role of Tax is widely unknown in myeloid cells, exposure of MDDC to recombinant Tax, lead to MDDC expression of activation and maturation markers [101, 102], and secretion of TNF-α, IL-12 and Mip1-β [102]. Whether exosome-containing Tax [97] are also able to modulate MDDC activation remains to be determined.

Moreover, Tax-induced MDDC activation also leads to T-cell stimulation and proliferation [102]. Inhibition of NF-κB pathways in Tax-exposed MDDC did not alter surface expression of activation markers, neither cytokine expression but reduced their ability to promote T-cell proliferation [102]. Thus, this suggests that Tax could also account for the strong inflammatory response and the ability of MDDC from HAM/TSP to induce T-cell proliferation. In contrast, following their transfer through nanotube or their expression in myeloid infected cells, the presence of p8/12 and 30 in monocytes and/or MDDC lead to impaired MDDC activation [37]. Thus, this could account for the reduced activity and lack of induction of T-cell proliferation observed in ATLL patients.

### pDC and IFN-α production

ATLL patients have impaired IFN-I production [31, 103], associated with decreased pDC populations [31, 104–106]. In addition, IFN-α impaired production and lower pDC count was associated with increased PVL in HTLV-1-infected donors [31, 103] suggesting a potential mechanism of evasion to IFN-I antiviral control (Table 1). Yet, viral burden might not be the sole mechanism for the blunted pDC IFN production, since viral DNA was evaluated as 400 to 4000 copies for 10,000 pDC, i.e. representing 4 to 40% of the total pDC population. This suggests that in ATL patients most pDC were free of viral DNA. Thus, even if HTLV-1 DNA presence in some pDC might directly impair their ability to produce IFN-I, other mechanisms are responsible for their lack of responsiveness in the absence of HTLV-1. Strikingly, pDC count was also decreased in HAM/TSP patients [104–106], even if IFN-induced signature was detected [107]. Transcriptomic analyses of PBMCs isolated from HAM/TSP blood samples evidenced an over-expression of a subset of IFN-stimulated genes. These genes are distinct from those induced during acute viral infection i.e. when IFN-I production is linked to viral control. Moreover, distinct IFN-I signature was not observed in asymptomatic carriers, in which viral replication is efficiently controlled, as demonstrated in vitro using recombinant IFN-α previously shown to control viral replication in T-cells [46, 47, 108], although not in MDDC [43]. This IFN-induced signature was mainly found in circulating monocytes and neutrophils from HAM/TSP patients but not in their T-cells, which are the cellular reservoir of HTLV-1 in vivo and thus are expected to be the main inducer as well as the main target of IFN. This IFN-inducible signature positively correlated with the clinical severity of the inflammatory disease but not with proviral load [107]. Altogether, these results might suggest that production of type I IFN (i.e. IFNα and β) by monocytes and neutrophils of HAM/TSP patients could be deleterious, while production by other cell types, such as stromal cells [108] from healthy carriers might control more efficiently the virus. However, the role of IFN-I production in patients and its role in disease progression or viral control remain elusive, as are the cells involved in its production in vivo.

In vitro studies, demonstrated that pDC purified from healthy donors produced important levels of IFN-I after exposure to highly concentrated cell-free HTLV-1 [109], and upon contact with infected cells containing viral biofilm [52]. Interestingly, side-by-side analyses demonstrated that purified biofilm triggered IFN-I pDC production whilst similar amount cell-free HTLV-1 preparation was less capable of having this effect [52]. Thus, as discussed above, viral biofilm might have been present in the cell-free HTLV-1 preparation used in the Colisson et al. study. Nonetheless, HTLV-1-induced pDCs response is dependent on TLR-7 signaling and involves mobilization of tumor-necrosis-factor related apoptosis inducing ligand (TRAIL) [52, 109], transforming them in IFN-producing killer pDCs (IKpDC) that are capable of inducing apoptosis in CD4^+^ T-cells expressing DR5, the TRAIL receptor [109]. Whether IKpDC persist during chronic infection and which role they might have in controlling HTLV-1 burden or disease progression remains to be investigated. Importantly, pDC responsiveness to infected cells was shown to be regulated by the composition of the extracellular matrix surrounding the viral biofilm [52], with in particular high density of terminal β-galactoside glycosylation leading to reduced pDC IFN-I production. Given that such glycosylation, also known as Tn antigen, is associated to the aggressiveness of cancer cells [110, 111], it might also control the ability of pDC to produce IFN-α in ATLL patients. Future investigations are required to clarify these points.

## Conclusion

Upon HTLV-1 infection, infected T-cells act as the viral reservoir until potential development, although in a small fraction of individuals, of an hyperproliferative blood malignancy, the ATLL or of a neuroinflammatory chronic disease known as HAM/TSP. However, even though both diseases are caused as a consequence of HTLV-1infection, the mechanisms leading to these two complete different disorders are fully unknown. Beside CD4^+^ and CD8^+^ T-cell infection, it is now nicely demonstrated that different subsets of the myeloid compartment are infected. In particular, both myeloid and monocyte-derived dendritic cells have been convincingly proved to be productively infected by HTLV-1 in vitro, and then able to transmit very efficiently HTLV-1 to T-cells. As for T-cells infection, DC infection relies on cell–cell contact and on viral biofilm accumulated at the surface of infected donor cells. However, the different DC subsets present in human mucosa or blood are not equally susceptible to HTLV-1 infection. Thus, it remains to be determined which mechanisms govern such restrictions. Especially because the cellular mechanisms controlling susceptibility *vs* resistance to HTLV-1 infection could be essential hints to prevent HTLV-1 dissemination. In contrast, whereas monocytes and pDCs do not support HTLV-1 infection in vitro, detection of viral DNA in theses subtypes in vivo has been a source of debates. This contradiction was recently removed by the discovery of HTLV-1-infected hematopoietic stem cells in the bone marrow of HAM/TSP patients. Thus, presence of viral DNA in monocytes and pDCs in vivo is very likely inherited from HSC during their differentiation, and monocytes or pDCs may not directly participate in viral dissemination during the primo-infection. Thus, while DC are accepted to be key players in viral dissemination during primo-infection, monocytes and pDCs might rather play an important role during the chronic phase allowing viral escape from the immune system and subsequent HTLV-1 associated diseases.

The complete characterization of HTLV-1-induced perturbations of the immune compartment is still lacking, in particular in understanding why the same virus can lead to opposite immune manifestation as immune tolerance leading to ATLL or chronic inflammation leading to HAM/TSP. Also, since the route of infection (breast-feeding, sexual intercourse or blood transfusion) might be a key factor in immune system maturation, and especially regarding the role of myeloid cells in controlling the viral adaptive immune responses, further investigations should be focused on understanding the role of myeloid cells in HTLV-1 spreading and disease progression.

## Data Availability

Not applicable

## Abbreviations

HTLV-1: Human T-cell leukemia virus type 1
ATLL: adult T-cell leukemia/lymphoma
HAM/TSP: HTLV-1-associated myelopathy/tropical spastic paraparesis
ACs: asymptomatic carriers
PVL: proviral load
myDC: myeloid dendritic cell
pDC: plasmacytoid dendritic cells
DC: dendritic cells
HSC: hematopoietic stem cells
MDDC: monocytes derived DC
IFN-I: type-I interferon
IL: interleukine
TGF: transforming growth factor beta
TNF-α: tumor necrosis factor alpha
AZT: zidovudine
TLR: toll-like receptor
MLV: murine leukemia virus
PBMCs: peripheral blood mononuclear cells
STING: stimulator of interferon genes
SAMHD1: SAM domain and HD domain contain protein 1
LTR: long terminal repeat
ECM: extracellular matrix
CNS: central nervous system
CCL5: chemokine (C–C motif) ligand
CXCL9: chemokine C-X-C motif ligand
CX3CR1: chemokine C-X3-C motif receptor
MHCI: major histocompatibility complex
NFκB: nuclear factor-kappa B
TRAIL: tumor-necrosis-factor related apoptosis inducing ligand
IKpDC: IFN-producing killer pDCs
